# The effect of hemoadsorption on rivaroxaban blood plasma concentration in emergency cardiac surgery

**DOI:** 10.1007/s12055-021-01183-4

**Published:** 2021-04-23

**Authors:** Bernard Krüger, Tobias Renner, Mathias Van Hemelrijck, Juri Sromicki, Ahmed Ouda, Carlos - A. Mestres

**Affiliations:** 1 Institute of Anesthesiology, University Hospital Zurich, Zurich, Switzerland; 2 Intensive Care Unit for Cardiovascular Surgery, University Hospital Zurich, Rämistrasse 100, CH-8091 Zurich, Switzerland; 3 Department of Cardiac Surgery, University Hospital Zurich, Zurich, Switzerland

**Keywords:** Hemoadsorption, Emergency cardiac surgery, Type A aortic dissection, Rivaroxaban, DOAC

## Abstract

Hemoadsorption was used in a 59-year-old patient with an acute type A aortic dissection, who was on rivaroxaban and dual antiplatelet therapy with clopidogrel and acetylsalicylic acid. Our aim was to expeditiously remove rivaroxaban preoperatively. After 8 h of hemoadsorption, the rivaroxaban blood plasma concentration (RBPC) did not decrease below 42.1 μg/l. Intraoperatively, hemoadsorption was repeated during extracorporeal circulation. Sixteen hours after surgery and a total of 13 h of hemoadsorption, the RBPC was 40.1 μg/l. Thereafter, the RBPC spontaneously decreased to 24.7 μg/l within 14 h. In our patient, hemoadsorption may have enhanced rivaroxaban removal at higher RBPC (cutoff value 40–50 μg/l). At lower RBPC, the removal of rivaroxaban may depend solely on the natural drug elimination process. The evolution of the RBPC under hemoadsorption in vivo warrants a thorough investigation. Further clinical studies are required to assess the effectiveness and limitations of hemoadsorption to preclude a fatal bleeding event in patients with rivaroxaban in need of major emergency surgery.

## Introduction

Direct oral anticoagulants (DOAC) and antiplatelet drugs may contribute to major bleeding in patients requiring emergency cardiac surgery [1]. For rivaroxaban and dabigatran, in urgent operations, it has been proposed to postpone surgery and monitor the drug concentration until a value of less or equal to 30 ng/ml is reached to reduce the hemorrhagic risk [2]. However, a more rapid approach in emergency surgery is required to save a patient’s life. Strategies to restore the hemostasis system include the removal of the active drug component [3]. Hemoadsorption—a technique similar to hemoperfusion—may be used for this approach. Blood is directed along polymer beads through a cartridge where hydrophobic molecules, up to a size of 55 kDa, are removed [4]. In vitro, hemoadsorption effectively reduced the plasma concentration of two DOACs and ticagrelor [5–7]. In vivo, hemoadsorption has been associated with reduced bleeding and transfusion requirements in surgical patients under rivaroxaban and ticagrelor [1]. However, the evolution of the rivaroxaban blood plasma concentration (RBPC) under hemoadsorption in vivo has not been thoroughly investigated yet.

We present a patient with an acute type A aortic dissection who was on rivaroxaban and dual antiplatelet therapy with clopidogrel and acetylsalicylic acid (ASA). Hemoadsorption was used to enhance the elimination of rivaroxaban and reduce the risk of a postoperative fatal bleeding event. Our aim is to report the course of RBPC under hemoadsorption in a relevant clinical scenario.

## Case report

A 59-year-old male patient, with a history of coronary artery disease, on 75 mg/day clopidogrel, and relapsing deep vein thrombosis, on 20 mg/day rivaroxaban, suddenly experienced chest pain and dyspnea. His left leg was concomitantly swollen and painful. After the onset of symptoms, he self-administered an additional dose of 20 mg rivaroxaban and called the emergency service. The patient received 500 mg ASA for potential acute coronary syndrome and was transported to the district hospital. The clinical examination revealed an edematous left lower extremity with no palpable femoral artery pulse, loss of sensibility, and reduced motor function. Computed tomography (CT) demonstrated an acute Stanford type A, DeBakey type I, aortic dissection with malperfusion of the inferior segment of the left kidney, and occlusion of the left common iliac artery (Penn class b). The patient was referred to our hospital for emergency surgery 7 h after the onset of symptoms. At hospital admission, neurologic symptoms were absent and vital signs were normal. Transthoracic echocardiography disclosed moderate aortic valve regurgitation and no pericardial effusion. Laboratory signs of acute organ dysfunction were absent (lactate 1.8 mmol/l, alanine transaminase 17 U/l, creatine kinase 188 U/l, creatinine 76 μmol/l). While the values of the activated partial thromboplastin time (aPTT) and prothrombin time (PT) were in the normal range with 31 s and 12.8 s, respectively, the international normalized ratio (INR) of the prothrombin time was mildly elevated with a value of 1.3. In contrast, a chromogenic anti-FXa assay calibrated for rivaroxaban (Biophen DiXal® test kit, Endotell AG, Allschwil, Switzerland) showed an RBPC value of 147.9 μg/l. To prevent aortic rupture and treat organ malperfusion, emergency central aortic repair was clearly indicated. However, it was felt that aortic repair, after the administration of 20 mg rivaroxaban 7 h ago, would entail an unacceptable risk of uncontrollable bleeding and eventual death. Because the patient was hemodynamically stable, it was decided to postpone surgery and treat symptomatic malperfusion of the left leg through an interventional approach. After pelvic angiogram, left femoral direct angiography, and invasive manometry, a membrane fenestration of the left external iliac artery was performed. To completely resolve malperfusion, the left external iliac and common femoral artery received percutaneous transluminal angioplasty (PTA) and stenting. The intervention was successful with no bleeding complications at the vascular access site and no reperfusion syndrome thereafter (maximum lactate value 3.7 mmol/l).

The patient was transferred to the intensive care unit (ICU) and connected to a veno-venous hemofilter (multiFiltrate CiCa®, Ci-Ca® Dialysat K2, Fresenius Medical Care, Bad Homburg, Germany; blood flow 150 ml/min, dialysate flow 3000 ml/h) equipped with a hemoadsorber (CytoSorb®, CytoSorbents Corporation, Monmouth Junction, NJ, USA; 300 ml). The purpose of hemoadsorption was to enhance rivaroxaban removal in addition to the natural elimination process. After 2.5 h, the RBPC decreased from 89.4 μg/l at ICU admission to 42.1 μg/l. Applying the institutional guidelines of the University Hospital Zurich, with an empirical cutoff RBPC value of 50 μg/l, the patient was cleared for surgery. While waiting for an operation theater to become available, the patient remained in the ICU under hemoadsorption. A follow-up measurement showed no further decrease of RBPC after a total of 8 h of hemoadsorption. A preoperative rotational thromboelastometry (ROTEM; ROTEM® delta, Tem Innovations GmbH, Munich, Germany) showed a normal result.

Nineteen hours after the onset of symptoms, the patient underwent ascending aorta replacement with open distal anastomosis under hypothermic arrest at 26 °C core temperature, using right subclavian artery and right atrial cannulation. Cardiopulmonary bypass (CPB; Stöckert S5, LivaNova, UK; CAPIOX® FX25-Oxygenator, Terumo, JPN), aortic cross-clamping, and antegrade cerebral perfusion times were 306, 120, and 71 min, respectively. A second hemoadsorber was used during CPB. The dissection was repaired using Teflon felt and surgical glue; the aorta was replaced by a 30-mm Dacron graft. CPB ultrafiltration was 8500 ml and Cell Saver® blood retransfusion was 1500 ml. The chest was left open because of right ventricular dysfunction, a distributive shock, and coagulopathy. Intraoperatively, the minimum thrombocyte count was 30 G/l and the maximum INR value 2.4, while ROTEM displayed a minimum FIBTEM MCF of 5 mm and no sign of hyperfibrinolysis in follow-up measurements. Coagulopathy was counteracted by transfusion of three units of fresh frozen plasma and four thrombocyte concentrates, and administration of 1000 IU prothrombin complex concentrate, 6 g fibrinogen, 500 IU factor VIII combined with 1200 IU von Willebrand factor, 1750 IU factor XIII, and 1500 mg tranexamic acid. Diffuse bleeding subsided 6 h after surgery. During surgery and the first 24 postoperative hours, a total of seven erythrocyte concentrates were transfused. The cumulative chest tube drainage 24 h after surgery was 1295 ml (54 ml/h average). RBPC follow-up measurements at 16 h and 30 h after surgery were 40.1 μg/l and 24.7 μg/l, respectively. Postoperatively, renal function deteriorated, but improved after postoperative day (POD) 3, without need for hemodialysis. The sternum was closed on POD 4, but endotracheal extubation was delayed until POD 11 because of the development of a ventilator-associated pneumonia and a mixed hypo- and hyperactive delirium. Electroencephalography and CT scan ruled out nonconvulsive status epilepticus and cerebral insult. On POD 13, the patient was transferred back to the referring institution. The total ICU and hospital length of stay were 26 days and 40 days, respectively. Eighteen months after surgery, our patient is alive and active, without impairing sequelae.

## Discussion

We report a patient who survived major emergency cardiac surgery without fatal bleeding despite preoperative treatment with rivaroxaban and dual antiplatelet therapy. Our strategy to control bleeding was to delay aortic surgery and first treat acute limb malperfusion interventionally, use hemoadsorption preoperatively to expedite rivaroxaban elimination, and counteract the irreversible effects of clopidogrel and ASA on platelets intraoperatively by platelet transfusion. As the result of our strategy, and despite a 5-h CPB time with associated consumption coagulopathy, the patient received multiple blood products and coagulation restoring factors; and in the end, it was possible to control bleeding after surgery.

Recently, Hassan et al. reported a lower bleeding and transfusion rate after emergency cardiac surgery in 39 patients on ticagrelor or rivaroxaban with intraoperative hemoadsorption during CPB, of whom only one was treated for acute aortic dissection in the ticagrelor group [1]. In addition, a recent case report described successful urgent off-pump coronary artery bypass surgery in a patient under ticagrelor and rivaroxaban by pre- and intraoperative use of a hemofilter equipped with CytoSorb® [8]. In none of these studies, the evolution of RBPC values under hemoadsorption was produced in detail.

**Fig. 1:**
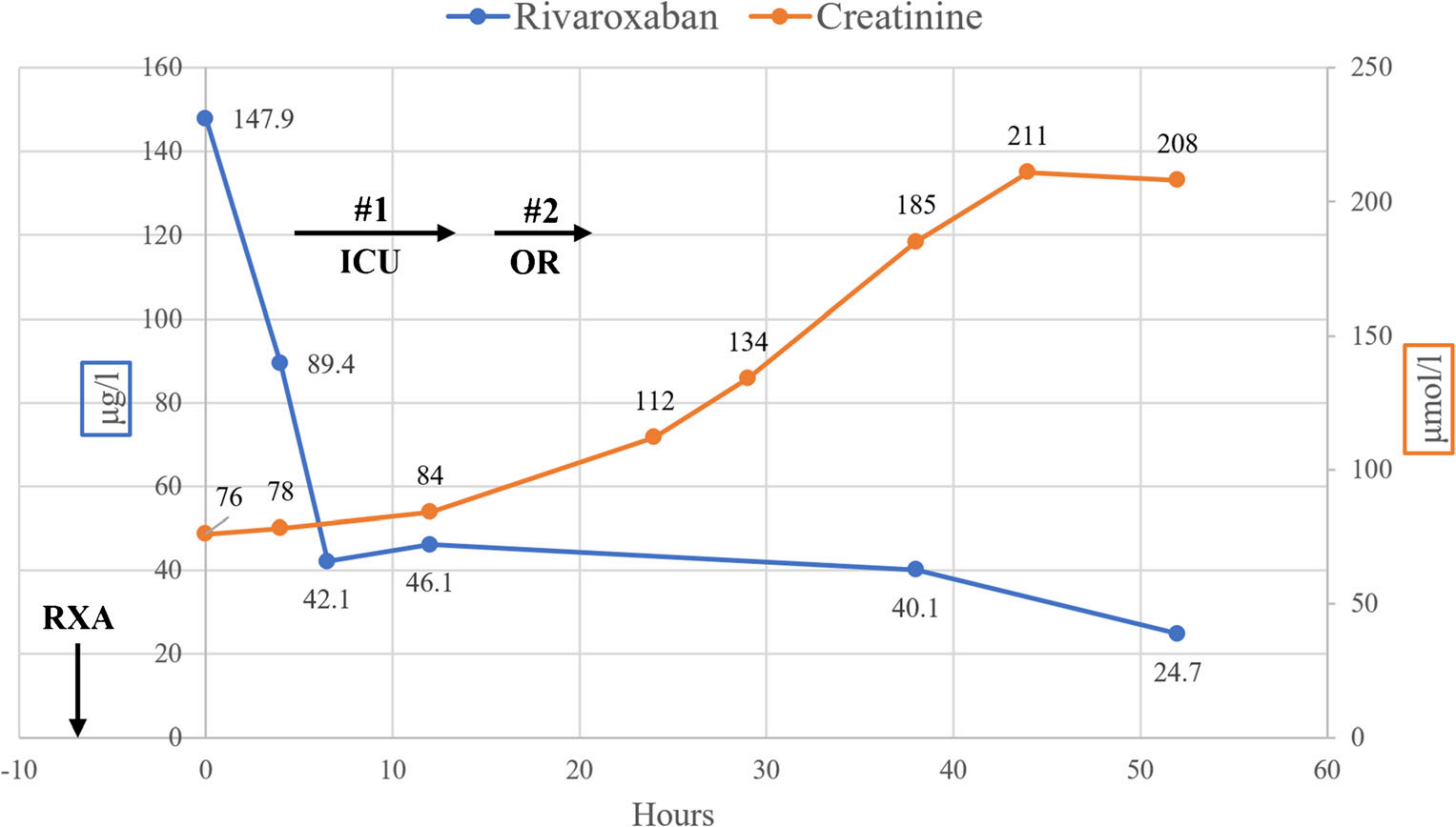
Evolution of rivaroxaban and creatinine blood plasma concentration over time. First values were measured at admission to our hospital—7 h after the administration of 20 mg rivaroxaban (RXA). #1 Hemoadsorption period preoperatively during intensive care (ICU). #2 Hemoadsorption period during surgery (OR)

Repeated measurements in our patient demonstrated that RBPC value decreased preoperatively from 147.9 μg/l at hospital admission to 89.4 μg/l spontaneously within 4 h and to 42.1 μg/l during hemoadsorption within 2.5 h. The evolution of the RBPC over time is displayed in Fig. 1. The measured values fall into the area of predicted rivaroxaban blood concentration between the “5th percentile” and a “typical patient aged 60 years,” described by Mueck et al. in a one-compartment model [9]. However, the course of measured RBPC values in our patient does not completely resemble the curves of the predicted values but shows a steeper course than predicted between 89.4 and 42.1 μg/l. This may signify that the elimination of rivaroxaban may have been enhanced by hemoadsorption during this period. The clearance effect of concomitant hemofiltration may be regarded as negligible as rivaroxaban is highly protein-bound in blood plasma and, hence, not considered dialyzable [9].

Continued hemoadsorption up to 13 h with two sequential adsorbers did not reduce the RBPC below a value of 40.1 μg/l in our patient. Furthermore, the RBPC increased from 42.1 to 46.1 μg/l during the first hemadsorption period (Fig. 1). In vitro, hemoadsorption removed rivaroxaban up to 91.6% with a steady plasma concentration < 50 μg/l after 1 h [6]. Hemoadsorption is considered to remove substances in a concentration-dependent manner [4]. Therefore, at lower RBPC, hemoadsorption may not add to the removal of rivaroxaban and the pharmacokinetics of rivaroxaban may depend solely on the natural drug elimination process in vivo. As approximately 30% of rivaroxaban is excreted by the kidneys unmetabolized [9], multifactorial perioperative renal dysfunction may explain the delayed RBPC decrease in our patient (Fig. 1).

Knowledge of the unique pharmacokinetic and pharmacodynamic (PK–PD) profile of a drug may represent a keystone for the successful implementation of hemadsorption into clinical practice. In addition to its chemical properties, rivaroxaban was the only coagulation-affecting drug in our patient that was amenable to removal by hemoadsorption, because of a plasma half-life of mean 12.08 (range 4.79–36.43) h and a reversible inhibitory effect on factor Xa [9]. In contrast, the PK–PD profiles of clopidogrel and ASA, with a plasma half-life of 6 h and 0.322 h respectively, and an irreversible inhibition of platelet aggregation for a platelet’s lifespan [10, 11] rendered hemoadsorption ineffective to counteract the action of these drugs. In our opinion, hemoadsorption in clinical practice may be most successful if used early after drug administration in selected drugs with appropriate chemical properties and a suitable PK–PD profile.

## Conclusion

Hemoadsorption may enhance rivaroxaban elimination above a RBPC of 40–50 μg/l. If used preoperatively, hemoadsorption may reduce the time of delay of emergency surgery. Prolonged use of hemoadsorption did not completely purify blood plasma from rivaroxaban in our patient. Further studies are required to establish the appropriate timing and duration of hemoadsorption in patients with rivaroxaban in need of major emergency surgery to assess its effectiveness and limitations in reducing postoperative fatal bleeding.
